# Mitogenomics of recombinant mitochondrial genomes of Baltic Sea *Mytilus* mussels

**DOI:** 10.1007/s00438-014-0888-3

**Published:** 2014-07-31

**Authors:** Małgorzata Zbawicka, Roman Wenne, Artur Burzyński

**Affiliations:** Department of Genetics and Marine Biotechnology, Institute of Oceanology, Polish Academy of Sciences, Powstańców Warszawy 55, 81-712 Sopot, Poland

**Keywords:** mtDNA recombination, D-loop, DUI, Evolution

## Abstract

Recombination in the control region (CR) of *Mytilus* mitochondrial DNA (mtDNA) was originally reported based on the relatively short, sequenced fragments of mitochondrial genomes. Recombination outside the CR has been reported recently with the suggestion that such processes are common in *Mytilus*. We have fully sequenced a set of 11 different mitochondrial haplotypes representing the high diversity of paternally inherited mitochondrial genomes of Baltic Sea *Mytilus* mussels, including the haplotype close to the native *Mytilus trossulus* mitochondrial genome, which was thought to have been entirely eliminated from this population. Phylogenetic and comparative analysis showed that the recombination is limited to the vicinity of the CR in all sequenced genomes. Coding sequence comparison indicated that all paternally inherited genomes showed increased accumulation of nonsynonymous substitutions, including the genomes which switched their transmission route very recently. The acquisition of certain CR sequences through recombination with highly divergent paternally inherited genomes seems to precede and favor the switch, but it is not a prerequisite for this process. Interspecies hybridization in the Baltic Sea during the recent 10,000 years created conditions for both structural and evolutionary mitochondrial instability which resulted in the observed variation and dynamics of mtDNA in Baltic Sea *Mytilus* mussels. In conclusion, the data shows that the effects of mitochondrial recombination are limited to the CR of few phylogenetic lineages.

**Electronic supplementary material:**

The online version of this article (doi:10.1007/s00438-014-0888-3) contains supplementary material, which is available to authorized users.

## Introduction

Mussels of the genus *Mytilus* have an unusual system of mitochondrial DNA (mtDNA) inheritance [referred to as doubly uniparental inheritance (DUI)], where the female type (F) is transmitted to all offspring and male type (M) only to the sons (Zouros et al. 1994; Skibinski et al. 1994). This system has also been observed in some other bivalve orders and families, e.g., Unionoida, Veneridae and Donacidae (Liu et al. 1996; Passamonti and Scali 2001; Curole and Kocher 2002; Serb and Lydeard 2003; Theologidis et al. 2008). Divergence between the F and M genomes can be greater than 40 %, but occasionally the M genome can be replaced by the F genome in a process called masculinization (Hoeh et al. 1997). In consequence, the divergence between paternally and maternally inherited genomes can be reduced. The well-documented examples of masculinization come from the Baltic population of *Mytilus trossulus* mussels. In this population the highly divergent M genome occurs very rarely and both genomes F and M are similar to the F genome of the congeneric *M. edulis*, and not to the native *M. trossulus* (Wenne and Skibinski 1995; Burzyński et al. 2003, 2006; Zbawicka et al. 2007). A hybrid zone around the Oresund and Danish belts separate Baltic *M. trossulus* from North sea *M. edulis.* Moreover, the Baltic population is composed of individuals of mixed genetic background (Riginos et al. 2002; Bierne et al. 2003; Kijewski et al. 2006, 2011). The hybrid zone apparently allowed complete asymmetric introgression of *M. edulis* F mtDNA (Rawson and Hilbish 1998; Quesada et al. 1999). The very young age of the Baltic Sea, together with the postglacial timing of *M. trossulus* invasion from the Pacific (Śmietanka et al. 2013), indicates that this process must have taken place during the last few thousands years. Despite that, all attempts to detect the relict native *M. trossulus* genomes in Baltic mussels failed, with one possible exception. Quesada et al. (2003) suggested the presence of the native *M. trossulus* M genome, but only a very short fragment of the genome was sequenced, precluding conclusive identification. Instability of mitochondrial genomes in the Baltic population was also exemplified by heteroplasmy for two and possibly three mitochondrial genomes of low divergence (Quesada et al. 2003; Zbawicka et al. 2003).

Another aspect of *Mytilus* mitochondrial genome instability is the apparent recombination signature, presented in the control region (CR) of multiple haplotypes (Burzyński et al. 2003, 2006; Rawson 2005; Venetis et al. 2007; Filipowicz et al. 2008). Baltic *M. trossulus* in particular have a great diversity of structural rearrangements in the CRs (Burzyński et al. 2003), which can be explained by duplication, deletion or intermolecular recombination (Burzyński et al. 2006). Moreover, its paternal lineage is dominated by mosaic haplotypes having *M. edulis* M-like CR segments, not present in maternally inherited haplotypes. It was hypothesized that the M-like fragment is necessary for a role reversal event (Zouros 2000; Burzyński et al. 2003; Cao et al. 2004). However, the discovery of genomes with mosaic CRs inherited maternally (Śmietanka et al. 2010) as well as the possibility that some masculinized genomes did not have the mosaic CRs (Burzynski et al. 2006) weakened the hypothesis.

Here we present, for the first time, the complete sequences of a representative set consisting of 11 mitochondrial genomes from Baltic *Mytilus*. Their comparative analysis sheds new light on the timing, mechanisms of emergence and evolution of recombinant mitochondrial genomes in bivalve species exhibiting the unusual system of DUI.

## Materials and methods

### Samples

A sample of 400 *Mytilus* sp. mussels collected from the Gulf of Gdańsk, southern Baltic and described previously (Burzyński et al. 2003, 2006) was used. The known taxonomic identity of the specimen, established using nuclear markers by Zbawicka et al. (2007), was typical for the Baltic hybrid population as defined by Kijewski et al. (2006) and Zbawicka et al. (2010). Representative mitochondrial haplotypes were selected for whole mitogenome sequencing, following the methodology described by Zbawicka et al. (2007). Nine haplotypes were derived from sperm, one from eggs and one from female somatic tissues. All haplotypes of *M. edulis* origin were genotyped at their CR first, using PCR, Southern hybridization and sequencing, as described by Burzyński et al. (2006) (Table 1). Identification of the F haplotype of *M. trossulus* origin was performed by PCR amplification of the mtDNA fragment spanning the 3’ part of *nad2* gene, two tRNA (*trnS* and *trnM*) and the 5′ part of *cox3* gene with a highly specific primer pair, F1T and U2T (Śmietanka et al. 2013). Initial screening of the whole sample revealed a few positive individuals, and the one with the strongest signal (female 62mc10) was selected. This female had two F haplotypes: one similar to *M. edulis* and the other similar to *M. trossulus*. Only the latter was analyzed. To ensure that no contamination influenced this unusual result, the following precautions were taken. All DNA work involving Baltic mussels was done in a separate laboratory, without any contact with mussels having native mtDNA of *M. trossulus*. Separate sets of equipment and reagents were used. Appropriate negative controls were included in all PCRs. Moreover, the resulting haplotype has unique structural characteristics, not seen in previously isolated genomes. It is therefore highly unlikely that it is a contamination product.

**Table 1:** Mitochondrial genomes sequenced during this study

ID	Sex	Haplogroup	Tissue	Total length	CR length	Accession number
178mc10	Female	*M. edulis* F	Eggs	16,745	1,158	KM192128
25mc10	Male	1a	Sperm	16,587	1,000	KM192131
107mc10	Male	1a	Sperm	16,593	1,006	KM192124
136mc10	Male	1a	Sperm	16,591	1,004	KM192126
152mc10	Male	1a	Sperm	16,589	1,002	KM192127
20mc10	Male	11a	Sperm	17,724	2,139	KM192130
115mc10	Male	11a	Sperm	17,720	2,134	KM192125
45mc10	Male	1	Sperm	16,583	996	KM192132
46mc10	Male	16	Sperm	>19,291	>3,704	KM192134
195mc10	Male	*M. edulis* M	Sperm	16,632	934	KM192129
62mc10	Female	*M. trossulus* F	Mantle	>17,472	>1,884	KM192133

### PCR and sequencing of mitochondrial DNA

The whole genomes were sequenced in two steps, as described previously (Zbawicka et al. 2007; Śmietanka et al. 2010). First, long-range (LR) PCRs were performed with highly specific primers. Then, overlapping fragments were re-amplified with nested universal primers and sequenced directly. The detailed information on primers used is given in Supplementary Table 1. Additional PCRs with primers covering the CRs were performed, as needed, to fill the gaps. For some haplotypes the structure of the CR was too complex. They contained large arrays of long repeats which were impossible to bridge with current sequencing technology. In such cases, we sequenced the whole coding part and as much of the CR as possible, using a combination of specific LR-PCR with re-amplification and direct sequencing.

For all LR-PCR, Phusion *Pfx* (Finnzymes Oy) polymerase was used according to the manufacturer’s protocol. For re-amplifications, a 1:800 dilution of the LR-PCR product was used as a template. All re-amplifications were performed as described previously (Zbawicka et al. 2007, 2010; Śmietanka et al. 2010). PCR amplifications were carried out in 15 μl reaction volumes containing 20 ng of template DNA, 0.4 μM of each primer, 200 μM nucleotides, 1.5 mM magnesium chloride, 0.5 unit of high-fidelity DyNAzymeEXT2 DNA polymerase (Finnzymes Oy) and appropriate reaction buffer from Finnzymes. All PCRs were performed in a T-gradient cycler from Biometra (Tampa, FL). PCR products (2 μl of each amplification) were visualized on 1 % agarose gels stained with ethidium bromide. PCR products were purified by alkaline phosphatase and exonuclease I treatment (Werle et al. 1994) and sequenced directly with BigDye™ terminator cycle sequencing method.

Sequence assembly and annotation followed the established protocol (Zbawicka et al. 2007, 2010; Śmietanka et al. 2010). The assembly was facilitated by Phred (Ewing et al. 1998) and performed in Gap4 (Bonfield et al. 1995; Staden 2001). De novo prediction of all protein-coding genes was attempted using a set of algorithms implemented in CRITICA (Badger and Olsen 1999), Glimmer3 (Delcher et al. 1999) and wise2 (Birney et al. 2004). For prediction of RNA genes, Arwen was used (Laslett and Canbäck 2008). All predictions were inspected and critically evaluated after comparison with the closest RefSeq annotations. The assembled and annotated sequences have been deposited in GenBank under accession numbers KM192124-KM192134.

### Bioinformatic analysis

For comparative analysis, 30 complete or nearly complete *Mytilus* mtDNA sequences were used. There were 19 sequences already present in GenBank (Table 2) and 11 newly obtained (Table 1). Individual gene sequences were extracted and aligned in MEGA5 (Tamura et al. 2011), using aminoacid translation as a guide. For most analyses the resulting alignments were concatenated. Genetic distance (*K*) based on Kimura’s two-parameter model (Kimura 1980) and divergence in synonymous (*K*_s_) and nonsynonymous (*K*_a_) substitutions, using modified Nei–Gojobori method (Nei and Gojobori 1986) with Jukes–Cantor correction, were calculated in MEGA5, with standard error (SE) computed over 1,000 bootstrap replicates. To identify the sites under selection, nonsynonymous and synonymous changes at individual codons were evaluated using several methods: single likelihood ancestor counting (SLAC), fixed effects likelihood (FEL), random effects likelihood (REL) (Kosakovsky Pond and Frost 2005b), fast, unconstrained Bayesian approximation (FUBAR) (Murrell et al. 2013) and mixed effects model of evolution (MEME) (Murrell et al. 2012), and GA-Branch (Pond and Frost 2005) in HyPhy (Scheffler et al. 2006), as implemented via the *datamonkey* web server (Kosakovsky Pond and Frost 2005a). Significance levels of 0.05 for SLAC and FEL and Bayes factor criterion of 50 for REL were used.

**Table 2:** Published genomes used in comparative analysis

Accession number	ID	Species	References
AY823625		*M. trossulus*	Breton et al. (2006)
AY823623		*M. trossulus*	Breton et al. (2006)
AY823624		*M. trossulus*	Breton et al. (2006)
FJ890849	azo20	*M. galloprovincialis*	Burzyński and Śmietanka (2009)
FJ890850	ori27	*M. galloprovincialis*	Burzyński and Śmietanka (2009)
NC_006161		*M. edulis*	Boore et al. (2004)
NC_015993		*M. californianus*	Cao et al. (2009)
EF434638	42ori	*M. galloprovincialis*	Filipowicz et al. (2008)
AY497292		*M. galloprovincialis*	Mizi et al. (2005)
AY363687		*M. galloprovincialis*	Mizi et al. (2005)
HM462080	kan12	*M. trossulus*	Śmietanka et al. (2010)
HM462081	kan35	*M. trossulus*	Śmietanka et al. (2010)
DQ399833		*M. galloprovincialis*	Venetis et al. (2007)
DQ198231	39mc10	*M. trossulus*	Zbawicka et al. (2007)
DQ198225	87mc10	*M. edulis*	Zbawicka et al. (2007)
GU936625	34LE	*M. trossulus*	Zbawicka et al. (2010)
GU936626	149LE	*M. trossulus*	Zbawicka et al. (2010)
GU936627	117LE	*M. trossulus*	Zbawicka et al. (2010)
JX486124		*M. californianus*	GenBank

To ascertain the recombination and identify recombination breakpoints, recombination detection algorithms: Geneconv (Padidam et al. 1999), MaxChi (Maynard Smith 1992), Chimaera (Posada and Crandall 2001), SiScan (Gibbs et al. 2000), Bootscan (Martin et al. 2005a) and 3SEQ (Boni et al. 2007), as implemented in RDP software (Martin et al. 2005b), were used. Only the recombination events detected with *p* < 0.05 after Bonferroni correction for multiple tests by at least two methods were considered. This seemed to be the optimal criterion for detecting recombination in this system.

## Results

### Mitochondrial genome organization

Complete sequences of nine and approximately 70–80 % of two more mtDNA haplotypes of Baltic *Mytilus* were obtained, including 100 % of coding sequence in all cases. They represent all haplogroups described previously from the Baltic (Burzyński et al. 2006) and a genome very similar (<1 % divergence) to the native *M. trossulus* F genome, not reported from the Baltic earlier (Table 1). The genomes have the same gene order as the published *M. edulis*, *M. galloprovincialis* and *M. trossulus* F and M genomes (Mizi et al. 2005; Breton et al. 2006; Zbawicka et al. 2007, 2010; Śmietanka et al. 2010). The annotated genes usually have very similar lengths to the related published genomes. Notably, the coding sequences of all haplotypes representing presumably masculinized genomes from 1a, 1 and 16 haplogroups have a length of 15,587 bp, the same as in the typical F genome of *M. edulis*. However, we noticed an interesting substitution in one of the 11a haplotypes (115mc10). The apparent substitution from T to C occurred in the stop codon of this gene, transforming the termination codon into CAA, encoding glutamine. The next possible stop codon is located 12 bp downstream, extending the *nad5* ORF to overlap the neighboring *nad6*.

There is a certain intra-group length polymorphism associated primarily with the CR. In the case of the four 1a haplotypes, the differences are limited to the CR (1,000–1,006 bp). The length of the single sequenced M haplotype differs by 2 bp from the previously published example of the Baltic *M. trossulus* M genome (Zbawicka et al. 2007) and this difference is also the result of a longer CR. Similarly, the length of two 11a haplogroup genomes is different (17,720 and 17,724 bp) and the difference is caused by different lengths of their CR (2,134 and 2,139 bp), but in this case there is also 1 bp difference in the length of the *lrrna* gene. In two cases, we were unable to sequence the CR because of the numerous and long repeats. The CR of one sequenced haplotype 16 (46mc10) was longer than 8 kb, as estimated by PCR products sizing. In the apparently native *M. trossulus* F-like genome (62mc10), the CR has a complex structure, with the functional *trnQ* gene translocated into the CR between its M-like and F-like parts, as described previously for other *M. trossulus* F genomes (Cao et al. 2009; Zbawicka et al. 2010; Śmietanka et al. 2010). However, this CR was also only partially sequenced (1,884 bp), because of the numerous and long repeats. The CR length exceeds 9 kb in this genome; it is 3× longer than in genomes derived from Scotland and Canada (Breton et al. 2006; Śmietanka et al. 2010; Zbawicka et al. 2010).

We propose one minor change in the annotations. In the new interpretation the *nad5* gene starts earlier, covering the region previously interpreted as UR5, with a small overlap with the *nad4L* gene. The *nad5* length change resulting from this reinterpretation is 21 bp in F-like and 36 bp in divergent M genomes.

### Recombination

**Fig. 1:**
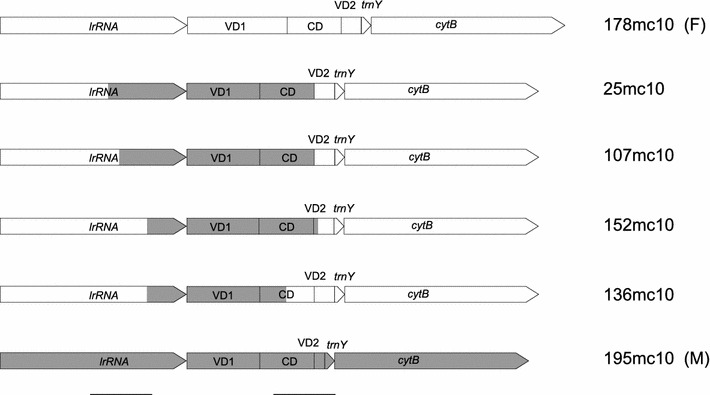
Schematic diagram of the CR structure of the four recombinant haplotypes from the 1a haplogroup (25mc10, 107mc10, 152mc10 and 136mc10). Typical F (178mc10) and M (195mc10) structures are shown for comparison. The *shaded boxes* represent regional similarities to the M genomes; *open boxes* represent F-related regions. All haplotypes were aligned at the *lrrna*. Alignments of the two regions containing recombination breakpoints (indicated by *vertical lines* below the figure) are presented in Fig. 2

**Fig. 2:**
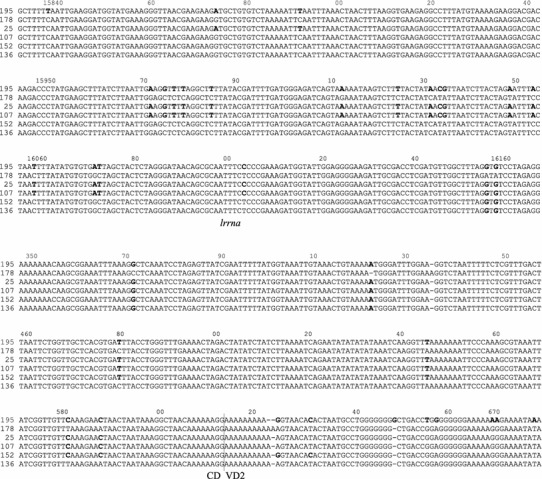
Alignment of the region of recombination of the six genomes shown in Fig. 1

**Fig. 3:**
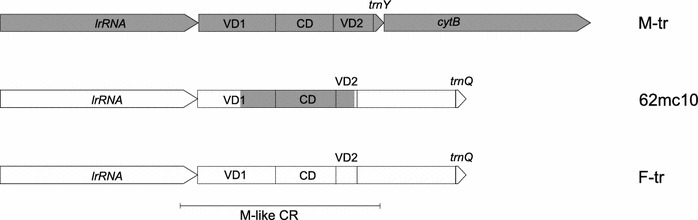
Schematic diagram of the CR of the native *M.* *trossulus* haplotype (62mc10). Typical *M. trossulus* M (GU936625, GU936626, HM462081), and F (AY823625, HM462080) genomes are shown for comparison. The *shaded boxes* represent regional similarities to the M genome; *open boxes* represent F-related regions. All haplotypes were aligned at the *lrrna*

The complete mtDNA sequences of six haplotypes: four 1a and one typical F and M have been aligned to elucidate the exact positions of recombination breakpoints. The recombination signal is very strong (Table 3). Surprisingly, the breakpoints detected in particular 1a genomes are not consistent. Two breakpoints are present in each of them, however, not necessarily at the same positions. The results are summarized in Fig. 1, and the alignment details can be seen in Fig. 2. The first breakpoint marking the change from F to M sequence is located in *lrrna* gene at three possible positions, while the second, marking the change from M to F sequence, is located within the CR, also at three different locations. In effect, the fragment with the high similarity to the M genome has different lengths in the four studied genomes: 1,388 bp (25mc10), 1,317 bp (107mc10), 1,150 bp (152mc10) and 934 bp (136mc10). The remaining parts of the 1a genomes show no signature of recombination and are derived from a typical F genome. Other sequenced genomes were also checked for the presence of recombination signatures. The mosaic or rearranged CR structure of haplotypes 11a, 1 and 16 described by Burzyński et al. (2006) was confirmed (data not shown). No recombination was detected in the coding parts of any of the sequenced genomes, unlike in the recent study of somatic recombination in *M. galloprovincalis* (Ladoukakis et al. 2011). However, new and unexpected signature of recombination was found in the CR of the native *M. trossulus* F genome (62mc10) when the sequenced part of the CR was compared with the corresponding part of the Canadian and Scottish genomes (accession numbers AY823625, HM462080, HM462081, GU936625, GU936626) (Table 3; Fig. 3). Apparently, a 706 bp long fragment, very similar in sequence to the native M genome of *M. trossulus*, replaced the corresponding part of the CR. This fragment has a distance of only 0.0129 from either 149LE or kan35—comparable to the distance between these M genomes and much bigger than the distance from the *M. trossulus* native F genomes (*K* = 0.1). This region has been previously shown to be derived from the ancient M genome (Rawson 2005), despite its presence in all native F genomes of *M. trossulus*. The two recombination breakpoints are located within this M-like part of the CR, making this a case of a secondary recombination: the new fragment of M genome is located within the old fragment of the M genome. The sequenced parts of this genome CR show further signs of sequence instability, such as a relatively big, 105 bp deletion in the 3′ part of the CR (VD2).

**Table 3:** Statistical support (average *p* value) given by recombination detection programs for recombination breakpoints in haplotypes from haplogroup 1a and in the native *M. trossulus* F genome from the Baltic Sea (62mc10)

Methods	1a	62mc10
RDP	1.649 × 10^−86^	2.538 × 10^−21^
GENECONV	9.873 × 10^−79^	1.268 × 10^−13^
Bootscan	3.175 × 10^−85^	3.025 × 10^−20^
MaxChi	6.591 × 10^−36^	1.778 × 10^−19^
Chimaera	5.480 × 10^−42^	4.454 × 10^−19^
SiScan	1.234 × 10^−47^	2.137 × 10^−18^
3Seq	1.006 × 10^−160^	4.794 × 10^−40^

**Fig. 4:**
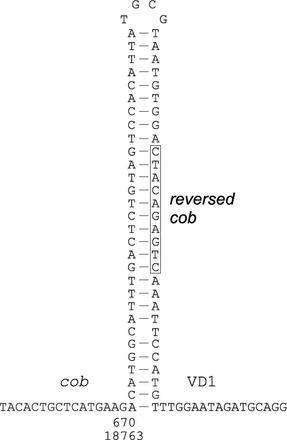
The fragment of the 46mc10 genome (haplogroup 16), with a *hairpin structure*, present near the two sequenced breakpoints. The *boxed* fragment is not derived directly from any of the parental sequences (VD1 or *cob*), but could be synthesized by strand switching during DNA synthesis only

The recombination breakpoints are usually located within fragments of high similarity between putative parental sequences precluding the exact identification of their position and context. However, in the case of the genome belonging to haplogroup 16 (46mc10), an interesting sequence was found at both recombination breakpoints. In this genome, no mosaic M–F structure was found, but an extensive array of repeats of apparently F origin is seen. The repeat array starts at VD1 of the CR and ends within the *cob* gene. At each of the two recovered breakpoints, the presence of the same structure was discovered (Fig. 4). It is formed by parts of the *cob* sequence and the short stretch of homology between VD1 and *cob*. The boxed fragment joining *cob* and VD1 is not homologous to either parental sequence, but it is perfectly matched to the 10 bp *cob* fragment upstream from the breakpoint, completing a very large hairpin structure around it with an overall stability of dG = −30 kcal/mol.

### Evolution

**Fig. 5:**
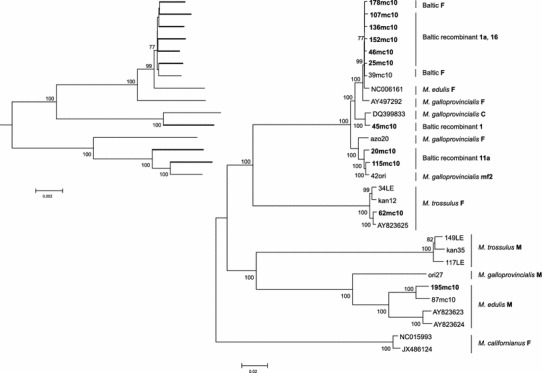
Neighbor-joining phylogeny based on concatenated protein-coding alignment (11,958 bp) from the whole mitochondrial genomes of *Mytilus* mussels. The genomes from the present study are in *bold*. Genome labels follow the convention outlined in Tables 1 and 2

To put the sequenced genomes in an evolutionary perspective, a neighbor-joining phylogenetic tree based on the concatenated protein-coding sequences was constructed (Fig. 5). The underlying genetic distances are listed in Supplementary Table 2. The distances separating any haplotype 16 (*K* = 0.0035, SE = 0.0006) or 1a (*K* = 0.0040, SE = 0.0006) from the F genomes (39mc10, 178mc10) are similar to the distance between these F genomes (*K* = 0.0039, SE = 0.0006). Slightly larger distances are observed between haplotypes from the recombinant 11a haplogroup and their sister nonrecombinant haplotype, the *M. galloprovincialis* F genome from Azov Sea (azo20)(*K* = 0.0163, SE = 0.0012). The native *M. trossulus* genome, while very distant from the other F-like genomes from the Baltic, shows only mildly biased affinity toward the published *M. trossulus* F genome from the West Atlantic (Breton et al. 2006) (*K* = 0.0046, SE = 0.0007), as compared to the genome from the East Pacific (Śmietanka et al. 2010) (*K* = 0.0114, SE = 0.0008), with an average distance within this group at 0.0087.

To assess the degree of selective pressure acting on genomes, the concatenated protein-coding sequences were analyzed in several comparisons. The genomes were sorted into groups consisting of 2–4 sequences and *K*_a_/*K*_s_ were calculated within groups. As expected, the *K*_a_ values are lower than *K*_s_, indicating purifying selection (Table 4). The values observed for the *M. edulis* M genome groups (average *K*_a_/*K*_s_ = 0.1241) are higher than for F genomes (*K*_a_/*K*_s_ = 0.0765), consistent with relaxed pressure in M groups, first postulated by Stewart et al. (1996). In the Baltic recombinants, this effect is even more pronounced, particularly for haplotypes from the groups 1a (*K*_a_/*K*_s_ = 0.2031) and 1 (*K*_a_/*K*_s_ = 0.2264). Interestingly, the recombinant 42ori genome compared with its closest nonrecombinant genomes revealed the degree of selective constraint similar to that of typical *M. edulis* or *M. galloprovincialis* F genomes. Contrary to that, both Baltic representatives of the 11a group did show elevated accumulation of nonsynonymous changes in comparisons involving the 42ori genome. The *M. trossulus* F genomes have relatively high *K*_a_/*K*_s_ (0.1605).

**Table 4:** *K*_a_/*K*_s_ comparisons within and between groups made of groups of 2–4 sequences of mitochondrial proteins

Comparison	Sequences used in each comparison	Origin of sequences	*K*_a_/*K*_s_
F genomes	M.edulis, Eur	39mc10, 178mc10	0.0765
	M. trossulus, Amer + Eur	AY823625, kan12, 34LE, 62mc10	0.1605
	M. galloprovincialis, Eur	azo20, AY497292	0.0512
M genomes	M.edulis, Eur	87mc10, 195mc10	0.0927
	M.edulis, Amer	AY823623, AY823624	0.1097
	M. galloprovincialis, Eur	ori27, AY363687	0.1381
	M. trossulus, Amer + Eur	kan35, 149LE, 117LE	0.1560
Recombinant genomes	1a, Baltic	25mc10, 107mc10, 136mc10, 152mc10	0.2031
	11a, Baltic	20mc10, 115mc10	0.0873
	1, M. galloprovincialis C	45mc10, DQ399833	0.2264
	11a and M. galloprovincialis mf2	20mc10, 42ori	0.0996
	11a and M. galloprovincialis mf2	115mc10, 42ori	0.0799
	1a, 16, Baltic	25mc10, 107mc10, 136mc10, 152mc10, 46mc10	0.1348
Recombinant and F genomes	1a, Baltic vs M.edulis, Eur	25mc10, 107mc10, 136mc10, 152mc10 vs 39mc10, 178mc10	0.1082
	16, Baltic vs M.edulis, Eur	46mc10 vs 39mc10, 178mc10	0.0907
	M. galloprovincialis mf2 vs M. galloprovincialis, Eur	42ori vs azo20, AY497292	0.0466
	11a, Baltic vs M. galloprovincialis, Eur	20mc10 vs azo20	0.0623
	11a, Baltic vs M. galloprovincialis, Eur	115mc10 vs azo20	0.0489

**Fig. 6:**
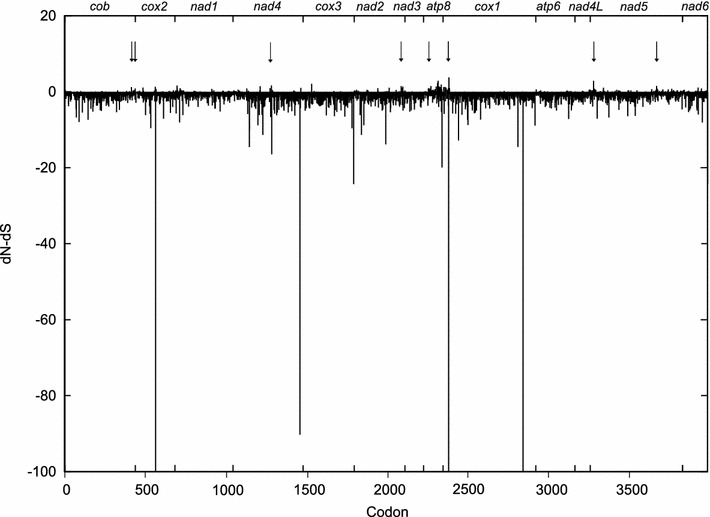
Concatenated protein-coding gene alignment was analyzed by FEL procedure in the context of the phylogenetic tree (Fig. 5).The d*N*–d*S* plot capped at −100 is presented. The *values above* *zero* indicate a candidate for positively selected sites. The gene position is shown at the *top*. The support for sites marked by *arrows* is presented in Table 5

To further elucidate the observed patterns a codon-by-codon analysis was applied to the concatenated data set. Out of 3,986 codons, 33 showed signature of positive and 3,253 of negative selection by at least one of the methods. An example of the dominance of negatively selected sites in FEL results is shown in Fig. 6. The sites with the strongest signatures of positive selection are marked by arrows and the support is summarized in Table 5. These nonsynonymous substitutions were, however, not distributed consistently on the phylogenetic tree; the GA-Branch analysis revealed accelerated accumulation of these substitutions at terminal branches only, particularly those leading to recombinant haplotypes (data not shown). We searched for group-specific substitutions using individual monophyletic groups or a single polyphyletic “group” identified simply by nature of their being recombinant. There were no substitutions common to any group of the recombinant genomes sequenced.

**Table 5:** Codons that may be under positive selection

Codon	Gene	*S*	NS	SLAC d*N*–d*S*	SLAC *p* value	FEL d*N*–d*S*	FEL *p* value	MEME *p* value	FUBAR Post. Pr.	FUBAR d*N*–d*S*
413	*cob*	0	7	0.31	0.09	1.217	0.040	0.078	0.669	0.236
436	*cob*	0	5	0.203	0.229	0.862	0.051	0.055	0.589	0.085
1272	*nad4*	2	8	0.205	0.253	0.940	0.431	0.019	0.534	0.225
2085	*nad2*	1	7	0.224	0.199	5.00 × 10^−9^	0.101	0.027	0.505	0.084
2254	*atp8*	0.75	5.25	0.192	0.235	0.912	0.084	0.025	0.499	0.022
2349	*cox1*	1	6	0.269	0.093	1.198	0.322	0.514	0.485	0.134
3278	*nad5*	3	9	0.285	0.148	2.891	0.11	0.138	0.926	2.511
3669	*nad5*	1	6	0.157	0.33	5.00 × 10^−9^	0.134	0.044	0.443	−0.009

*S* observed synonymous changes, *NS* observed nonsynonymous changes

## Discussion

The repertoire of mitochondrial genomes present in the paternal lineage of the Baltic *Mytilus* population is surprising. They represent all four major F lineages present in the *M. edulis* species complex (Śmietanka et al. 2009, 2010, 2013). The recent advances in transcriptomics (Chatzoglou et al. 2013) allow more precise annotation of some protein-coding genes within this diverse data set. Our proposal to extend the *nad5* gene is consistent with the notion that polycistronic transcript consisting of *nad4l*, *nad5*, and *nad6* is expressed in *Mytilus* (Chatzoglou et al. 2013; Sańko and Burzyński 2014). The fragment we propose to include as coding is present within the transcript. The only drawback of this proposal is the generated overlap between *nad4* *l* and *nad5.* We argue that it is quite possible to have overlapping genes within such a transcript. The extension in *nad5* ORF of the 115mc10 genome leads to another example of gene overlap, yet in this case it cannot be avoided by changing annotations.

### Native *M. trossulus* genome in the Baltic Sea

We demonstrate for the first time the presence of the native *M. trossulus* mtDNA in the Baltic Sea. The haplotype C2 (T-5) reported by Quesada et al. (2003) from Baltic Sea and suggested to be the ancestral European *M. trossulus* M genomes is in fact most likely one of the recombinant 1a haplotypes, with the first breakpoint at the same positions as in the 107mc10 genome. The overall distance between T-5 and 107mc10 over the whole published sequence is only *K* = 0.0058 (SE = 0.0041), much lower than its distance from the native *M. trossulus* genomes (*K* = 0.0397, SE = 0.0167). Contrary to that, the 62mc10 genome we report here is most certainly closely related to the native *M. trossulus* F genomes (Fig. 1). The possibility that this genome is an artifact is unlikely for the following reasons. We took utmost care to avoid contamination; therefore, we believe that the obtained sequences are present in the studied animal. The presence of *numts* in Baltic Sea mussels was negatively verified (Zbawicka et al. 2007), and given the technique used to obtain the sequences it is extremely unlikely that such long parts of mtDNA could be derived from the nuclear genome. The recombinant structure of the CR of this genome is also unlikely to be a product of somatic recombination, because no parental sequences were found in this individual. Interestingly, 62mc10 was a heteroplasmic female. There was no indication of the biased presence of such genomes in males, but the mechanism proposed for this type of heteroplasmy (two divergent genomes in a female) ultimately involves the inheritance of one of the genomes through the paternal lineage in the female ancestor (Obata et al. 2006, 2007; Kyriakou et al. 2010). Therefore we cannot be sure what the transmission route of this genome is, but it certainly is currently very rare in the Baltic Sea. Despite that, it is clearly the part of the same radiation as the other fully sequenced F genomes of *M. trossulus* (Fig. 1). Since it has been shown that this radiation took place post-glacially (Śmietanka et al. 2013), we can conclude that the age of this genome is in the order of 10^4^ years.

### Recombination mechanism

The strong support for the secondary recombination within the 62mc10 genome was unexpected. We have not found the parental native *M. trossulus* M genome in the Baltic, but the fragment is almost 10× more similar to the contemporary M genomes from other *M. trossulus* populations than to the respective fragment of the F genomes implying that the native M genome must have also been present. The alternative scenario with the introgression of the genome from some other population in Scandinavia or Scotland can be also considered but will require further screening for similar rearrangements in these populations. Since the same part of the molecule was shown to be involved in a similar recombination event (Rawson 2005; Breton et al. 2006; Cao et al. 2009; Śmietanka et al. 2010; Zbawicka et al. 2010), and no other part of the molecule seems to have any evidence of recombination, it is tempting to speculate that the propensity of the CR to undergo recombination is the driving force of these events. More support for this notion comes from the analysis of recombination breakpoints in the representative haplotype from the 16 haplogroup (46 mc10). This haplogroup was originally considered very young and we speculated that the genomes belonging to this haplogroup could have originated within the individuals we found them in (Burzyński et al. 2006). Given the number of nonsynonymous polymorphisms the sequenced genome accumulated, it seems rather unlikely. However, this genome must still be considered very young, since its distances from its nearest non-rearranged relatives are very small (Fig. 5). We hope that the structure of the breakpoints within this genome are close to their primary, nondegenerated state. The very stable hairpin structure found at the breakpoint has twofold consequences. Such structures by themselves are considered recombinogenic (Chen 2013) and could contribute to the development of long arrays of repeats within the CR of this genome. On the other hand, the sequence at the breakpoint (boxed in Fig. 2) could not possibly be obtained from either of the potential parental fragments. Instead, it is complementary to the fragment of the truncated *cob* on the other side of the hairpin. The only possibility of obtaining such a fragment is to synthesize it using wrong strand as a matrix for DNA synthesis. Although not conclusive, it suggests the involvement of DNA replication in these rearrangements. Short tandem repeats capable of forming secondary structures are very common in animal mtDNA genomes. These can easily form tandem arrays of several hundred to over a thousand nucleotides (He et al. 2011).

### Role reversal and recombinantion

A connection between recombination and masculinization was first hypothesized upon the discovery of CR recombinants (Burzyński et al. 2003). It was suggested that the M-derived fragment of the CR inserted into the otherwise F-like genome caused it to invade the paternal route of inheritance. This hypothesis was formulated based on a limited set of sequence information, covering only the CR and short flanking coding sequences. Mild objections have been raised on the grounds that other parts of these genomes may also have been mosaic (Venetis et al. 2007). With the complete sequences of the haplotypes in question, we can now be sure that recombination in their case is indeed limited to the short part of the CR. It has also been pointed out that the proof for the paternal inheritance of recombinant genomes was indirect only (Cao et al. 2009). The apparently increased rate with which these genomes accumulate nonsynonymous substitutions now provides further argument for their paternal inheritance. The distribution of this feature across the phylogenetic tree (Fig. 5; Table 4) shows that the recombination events predate masculinization. There are two phylogenetic clades hosting the majority of masculinized haplotypes from the Baltic (Fig. 5). The older clade is closely related to the *M. galloprovincialis* F genome (azo20). It has been dated at approximately 300 KYA (Śmietanka et al. 2010). The two masculinized haplotypes from the Baltic (20mc10 and 115mc10), belonging to the 11a haplogroup (Burzyński et al. 2006) and one recombinant but apparently nonmasculinized haplotype from the Mediterranean Sea (42ori)(Filipowicz et al. 2008) form this well-resolved clade. The two Baltic haplotypes must have independently switched their transmission routes to account for the observed pattern of polymorphism (Table 4). This pattern is consistent with the scenario under which first a recombination event occurs, then the recombinant genome exists in the population for a while (inherited maternally), and later it becomes masculinized and is thereafter inherited through males. Therefore, the masculinization is likely more recent within this clade than the clade age. The distance between the 115mc10 and 42ori haplotypes is 3× lower than the distance from their common nonrecombinant sister haplotype (azo20). It can be concluded that each of the haplotypes, 20mc10 and 115mc10, independently experienced a role reversal (masculinization) event. The alternative interpretation, involving one masculinization event of the ancestral genome to 115mc10 and 20mc10 followed by a feminization event in the formation of 42ori, is equally parsimonious, but would not lead to the observed patterns of nonsynonymous substitutions. The second clade containing haplotypes from 1a haplogroup contains not only recombinant, but also typical F haplotypes. The clade is internally unresolved (Fig. 5; Supplementary Fig. S1–S3) and, given the fact that full mtDNA sequences were used to construct the tree, there is not enough phylogenetic information accumulated within this clade to ever resolve the relationships between haplotypes. The only plausible cause for this is the extremely young age of this group. In our earlier work (Burzyński et al. 2003, 2006), haplotypes from this group were assumed to be a product of a single recombination event, but the first recombination breakpoint was not accounted for. Given the now resolved positions of all the breakpoints (Figs. 1, 2), this assumption is no longer valid. To explain the different extent of the M-like fragment present in the CR of haplotypes from the 1a haplogroup, we must assume a series of homologous recombinations, either independently involving the highly divergent M genome or, what is more likely, sequentially involving the primary recombinants and typical F genomes. In either case these events must be very recent indeed, the divergence between the genomes in question does not exceed a few substitutions (Supplementary Table 2, *K* < 0.005) and is consistent with the history of this clade confined entirely to the short lifespan of the Baltic Sea (no more than 10,000 years).

### Hybridization and masculinization

In the hybrid population of Baltic *Mytilus* mussels, apparently high level of both CR recombination and masculinization has been taking place in the last few thousands years. A possible explanation could involve the destabilization of mtDNA due to cytonuclear incompatibility. The exclusive presence of *M. edulis* mtDNA in Baltic Sea *Mytilus* mussels would not cause functional difficulties if the nuclear background was also dominated by *M. edulis* component. Several papers suggested this to be the case (Riginos et al. 2002; Bierne et al. 2003; Kijewski et al. 2006, 2011). However, with more nuclear markers it becomes apparent that the Baltic Sea mussels are much closer to *M. trossulus* than *M. edulis* (Zbawicka et al. 2014). Therefore, the hybridization could have caused abrupt and rapid increase in the M genome incompatibility, favoring masculinization. Given the presented pattern of polymorphisms, it is tempting the speculate that preexisting recombinant genomes (such as 20mc10 and 115mc10) become masculinized only during the recent history of the Baltic Sea, together with the newly generated recombinants (1a haplotypes). Apparently, the genomes with M-like CRs are preferentially masculinized, but the existence of masculinized genomes with complex CR structures but without M-like sequences (such as 46mc10) suggests that these genomes may spread due to factors other than the recognition of particular sequences within the CR.

## Electronic supplementary material

Below is the link to the electronic supplementary material.

Supplementary material 1 (PDF 109 kb)

Supplementary material 2 (PDF 71 kb)

Supplementary material 3 (PDF 102 kb)
