# Supplementary material for: Mitogenomics of recombinant mitochondrial genomes of Baltic Sea Mytilus mussels
Source: Mol Genet Genomics. 2014 Jul 31;289(6):1275–87. doi: 10.1007/s00438-014-0888-3 (PMC4236608; doi:10.1007/s00438-014-0888-3)
Supplement: Supplementary file 1 — Supplementary material 1 (PDF 109 kb) [file 438_2014_888_MOESM1_ESM.pdf]

Supplementary Table 1
Information on PCR primers used in mtDNA sequencing.

Name	Location	Strand	Haplotype	Annealing	Sequence
AB44	*nad3*	F	e, M (11a, 1, F, 16)	58-66°C	GAATACCGTGAAGGTTCTTTGGAGTG
AB45	CD	R	M, (M, 1a)	58-66°C	AGGCAGCCCTTTTTATGAGTTGATC
AB46	CD	R	M (11a)	60°C	AGGCAGCCCTTTATATGAGTTTACAGC
AB46R	CD	F	M (11a)	60°C	GCTGTAAACTCATATAAAGGGCTGCCT
AB47	CD	R	M (1)	58°C	TTGGGTAGCCTTATTTTTAGGAGTTTACAG
AB47R	CD	F	M (1)	55°C	CTGTAAACTCCTAAAAATAAGGCTACCCAA
AB48R	CD	F	M, (M, 1a)	62°C	TCTACACGCTTAACTTCCCTGCCA
AB49	CD	R	e (F)	59°C	CACACTTAACAACCTATCACCAAGCAA
AB49R	CD	F	e (F)	59°C	TTGCTTGGTGATAGGTTGTTAAGTGTG
AB50R	*cox1*	R	e, M (11a, 1, F, 16)	55-66°C	AAGACACCCAATGCCAATGAGC
AB51Rv2	CD	R	16	59°C	AAAGGGGTGAAATTCCATGTTTGG
AB51v2	CD	F	16	59°C	CCAAACATGGAATTTCACCCCTTT
TRO7	CD	R	t	62°C	TTTGCCTGTTACTAGCACAAATCCTT
TRO10	CD	F	t	62°C	GCAGAGAAAGTGGTGAATTTGACC
LSS1	*lrrna*	F	e, t, M	61°C	GGATAGAAACTGAACTAGCT
LSS1R	*trn I*	R	e, t, M	61°C	ATGATCCAGTTGCTTTG
LSS2	*lrrna*	F	e, t, M	61°C	TTGTTAAACAGTCGAGAAG
LSS2R	*srrna*	R	e, t, M	61°C	TGGTTGTTTACTCTAATATGG
LSS3	*srrna*	F	e, M	52°C	TAGCAACAACTTCCGTT
LSS3f	*srrna*	F	t	52°C	ACTTCCGTTACTGCTACC
LSS3R	*nad6*	R	e	52°C	TGTTCTTAGTATTTATCCTAATG
LSS3Rf	*nad6*	R	t	52°C	TATGTTTTAAGGATCTACCC
LSS3Rm	*nad6*	R	M	52°C	TGTACTAAGAATTTACCCTAAC
LSS4	*srrna*	F	e, t, M	56°C	GATAACAGGGTATCTAATCC
LSS4R	*nad5*	R	e, t, M	56°C	TATAGGGCACGAATTGT
LSS5	*nad6*	F	e, M	52°C	TCATTAGGATAAATACTAAGAAC
LSS5f	*nad6*	F	t	52°C	GGGTAGATCCTTAAAACATA
LSS5R	*nad5*	R	e, M	52°C	AAATAACAAAAGACTATCTGC
LSS5Rf	*nad5*	R	t	52°C	ACAATAAGAGGCTATCTGC
LSS6	*nad5*	F	e, t, M	61°C	ACAATTCGTGCCCTATA
LSS6R	*trn T*	R	e, t, M	61°C	GCCTTGTAAGTCGTAGAA
LSS7	*nad5*	F	e, M	61°C	GCAGATAGTCTTTTGTTATTT
LSS7f	*nad5*	F	t	52°C	GCAGATAGCCTCTTATTGT
LSS7R	*atp6*	R	e, t, M	61°C	TTTCTAGATTTGATGCTCA
LSS8	*atp6*	F	e, t, M	63°C	AATGTAACACTGAATACAAGC
LSS8R	*cox1*	R	e, t, M	63°C	ATAAGAAATGGAGAAAAGC
LSS9	*atp6*	F	e, t, M	52°C	TGAGCATCAAATCTAGAAA
LSS9R	*cox1*	R	e, t, M	52°C	CGAGCTGAGCTTTATGT
LSS10	*cox1*	F	e, t	63°C	AAGAAGACACCCAATGC
LSS10m	*cox1*	F	M	56°C	AAATGACACAGCAGACC
LSS10R	UR4	R	e, t, M	56-63°C	GGAAGTGTACTGTGTGTATTT
RSS1	*trn Y*	F	e, t, M	56°C	CCTTTCTTATGAGATGGT
RSS1R	*cox2*	R	e, t, M	56°C	ATCACCAAAATATCGACT
RSS2	*cob*	F	e, t, M	54°C	TATAAAAACGCCTATTCA
RSS2R	*trn L1* (CUN)	R	e, t, M	54°C	CGAGCTTAAATCGTATG
RSS3	*cox2*	F	e, t, M	56°C	AGTCGATATTTTGGTGAT
RSS3R	*nad1*	R	e, t, M	56°C	CCCGATTAGTCTCAGC
RSS4	*trn L1* (CUN)	F	e, t, M	56-61°C	ATATTCTTGTTTGTGTGTGAAT
RSS4R	*nad4*	R	e, t	56°C	CCTCCTCACCCTAAACC
RSS4Rm	*nad4*	R	M	61°C	ACCTTCTCACCCTAAAGC
RSS5	*nad1*	F	e, t	59°C	CTGAGACTAATCGGGC
RSS5m	*nad1*	F	M	59°C	CTGAAACTAATCGGGCA
RSS5R	*cox3*	R	e, t	59°C	ACGAGAATAAGGATTACG
RSS5Rm	*cox3*	R	M	59°C	CGGGAATAAGGATTACG
RSS6	*nad4*	F	e, t	54°C	CGAAAGTGTGTTAGCG

RSS6m	*nad4*	F	M	52°C	TTTTGAAAGGGTGTTAG
RSS6R	*cox3*	R	e, t, M	54°C	TGGGCTTGAGTTACAA
RSS7	*cox3*	F	e, t, M	54°C	CTCGTTACTATGTACCAGG
RSS7R	*trn M*	R	e, t, M	54°C	AATCATAGGTCTTCTGTCC
RSS8	*cox3*	F	e, t, M	54°C	TTTGTAACTCAAGCCCA
RSS8R	*trn R*	R	e, t, M	54°C	ACCCACTCTTTTTGAGC
RSS9	*trn S2* (UCN)	F	e, t, M	53°C	AATCAAGTACCAAAGCG
RSS9Rv2	*nad3*	R	M	53°C	CAAAGAACCTTCACGG
RSS9Rv3	*nad3*	R	e, t	53°C	CGCATAAATGCCACTT
RSS10R	*cox1*	R	e, t	61°C	ACATAAAGCTCAGCTCG
RSS10Rm	*cox1*	R	M	61°C	GCTCAGCTCGCTCC
RSS10v2	*trn S1* (AGN)	F	e, t, M	61°C	ATAATAGGGCTGCTAACTT
AB15	*lrrna*	F	e, t, M	61-66°C	TTGCGACCTCGATGTTGG
AB16	CD	R	e, t, M	66°C	CAGGCTATAGAGCATAATCTAAAACG
AB25	VD1	F	M	61°C	CGCTTAACTTCCCTGCCA
AB26	VD1	R	M	61°C	TCTAAAACGAGGTATGG
AB28	*cob*	R	e, t, M	61°C	TGTACTTCGTCACGGACCAAC
AB32	CD	F	e, t, M	66°C	TGTCAGAGTCATGTGAGACTTAACC
AB40	*lrrna*	F	e, t, M	52°C	GACGACAAGACCCTATGAAGC
AB52	*lrrna*	F	e, t, M	60°C	CGACTGTTTAACAAAAACATTTCC
CBM1	*lrrna*	F	e, t, M	56°C	AGAACGGCGTGAGCTAGTTC
CBM2	*cob*	R	e, t, M	56°C	ACCTTCACCAGGCGTTTAAG
CBM9	CD	R	e, t, M	52°C	ACGTGAGCAACCAGAA
TRO3	VD1	F	t	54°C	GTAAGCATTTTTCAGAGTCA
TRO4	*lrrna*	R	t	54°C	ATTATTTATGTCATTACAGATCC
TRO6	*cob*	R	t	56°C	CATAGAGGACCCATTTGC
F1T	*cox3*	F	t	58°C	TTCCTAGTGCAACTTCGAGAATA
U2T	*nad2*	R	t	58°C	AAGGAAAGGAGGCATCCC

M - *M. edulis* M genome specific, e - *M. edulis* F genome specific, t - *M. trossulus* F genome specific.
Long range primers used in amplification of coding part are located above the line.
