# Supplementary material for: Mitogenomics of recombinant mitochondrial genomes of Baltic Sea Mytilus mussels
Source: Mol Genet Genomics. 2014 Jul 31;289(6):1275–87. doi: 10.1007/s00438-014-0888-3 (PMC4236608; doi:10.1007/s00438-014-0888-3)
Supplement: Supplementary file 2 — Supplementary material 2 (PDF 71 kb) [file 438_2014_888_MOESM2_ESM.pdf]

Supplementary Table 2

Genetic distance (K) based on Kimura's two-parameter model calculated for concatened proteins sequences 29 study genomes.

	195mc10	87mc10	ori27	AY823623	AY823624	149LE	117LE	kan35	34LE	62mc10tr	kan12	AY823625	NC006161	AY497292	azo20	DQ399833	39mc10	178mc10	25mc10	107mc10	136mc10	152mc10	20mc10	115mc10	42ori	45mc10	46mc10	NC015993	JX486124
195mc10		0.0016	0.0033	0.0021	0.0023	0.0062	0.0058	0.0061	0.0052	0.0050	0.0054	0.0051	0.0050	0.0050	0.0051	0.0048	0.0050	0.0052	0.0051	0.0051	0.0051	0.0052	0.0052	0.0051	0.0051	0.0052	0.0050	0.0068	0.0068
87mc10	0.0205		0.0035	0.0021	0.0022	0.0059	0.0056	0.0058	0.0051	0.0050	0.0053	0.0050	0.0046	0.0045	0.0047	0.0047	0.0044	0.0046	0.0045	0.0045	0.0046	0.0046	0.0047	0.0047	0.0046	0.0047	0.0045	0.0068	0.0070
ori27	0.1221	0.1211		0.0034	0.0035	0.0065	0.0058	0.0063	0.0056	0.0052	0.0058	0.0055	0.0049	0.0050	0.0053	0.0051	0.0050	0.0051	0.0050	0.0051	0.0051	0.0051	0.0057	0.0056	0.0055	0.0055	0.0051	0.0068	0.0068
AY823623	0.0647	0.0637	0.1137		0.0011	0.0061	0.0054	0.0060	0.0050	0.0051	0.0051	0.0050	0.0038	0.0037	0.0042	0.0037	0.0038	0.0039	0.0037	0.0039	0.0039	0.0039	0.0044	0.0043	0.0043	0.0039	0.0038	0.0065	0.0065
AY823624	0.0661	0.0650	0.1151	0.0134		0.0061	0.0053	0.0059	0.0048	0.0049	0.0049	0.0047	0.0037	0.0036	0.0041	0.0038	0.0036	0.0037	0.0037	0.0037	0.0037	0.0037	0.0043	0.0044	0.0044	0.0040	0.0036	0.0067	0.0067
149LE	0.2799	0.2807	0.2775	0.2817	0.2827		0.0011	0.0011	0.0051	0.0052	0.0053	0.0054	0.0061	0.0057	0.0059	0.0062	0.0060	0.0060	0.0059	0.0059	0.0061	0.0060	0.0057	0.0055	0.0056	0.0062	0.0061	0.0051	0.0052
117LE	0.2822	0.2818	0.2786	0.2824	0.2841	0.0158		0.0011	0.0051	0.0054	0.0053	0.0056	0.0060	0.0056	0.0058	0.0062	0.0058	0.0058	0.0057	0.0057	0.0059	0.0059	0.0058	0.0054	0.0056	0.0062	0.0059	0.0049	0.0049
kan35	0.2825	0.2826	0.2783	0.2833	0.2845	0.0134	0.0156		0.0051	0.0053	0.0052	0.0055	0.0063	0.0059	0.0061	0.0064	0.0062	0.0064	0.0061	0.0062	0.0064	0.0063	0.0060	0.0058	0.0059	0.0065	0.0063	0.0051	0.0051
34LE	0.2814	0.2811	0.2776	0.2858	0.2872	0.2809	0.2794	0.2811		0.0010	0.0006	0.0008	0.0038	0.0034	0.0041	0.0039	0.0036	0.0037	0.0038	0.0036	0.0038	0.0034	0.0038	0.0038	0.0039	0.0039	0.0037	0.0048	0.0045
62mc10tr	0.2828	0.2813	0.2799	0.2867	0.2884	0.2820	0.2811	0.2828	0.0113		0.0010	0.0006	0.0035	0.0032	0.0037	0.0036	0.0034	0.0035	0.0035	0.0034	0.0036	0.0032	0.0034	0.0035	0.0035	0.0037	0.0034	0.0045	0.0042
kan12	0.2821	0.2812	0.2774	0.2850	0.2867	0.2809	0.2796	0.2811	0.0066	0.0114		0.0009	0.0036	0.0032	0.0037	0.0037	0.0034	0.0035	0.0035	0.0034	0.0036	0.0032	0.0035	0.0035	0.0036	0.0037	0.0034	0.0048	0.0045
AY823625	0.2821	0.2806	0.2789	0.2858	0.2878	0.2811	0.2801	0.2818	0.0082	0.0046	0.0085		0.0037	0.0034	0.0040	0.0038	0.0036	0.0037	0.0037	0.0036	0.0037	0.0033	0.0036	0.0036	0.0037	0.0039	0.0036	0.0048	0.0045
NC006161	0.2660	0.2672	0.2653	0.2688	0.2700	0.2783	0.2802	0.2800	0.1872	0.1868	0.1869	0.1866		0.0009	0.0015	0.0014	0.0006	0.0007	0.0006	0.0008	0.0006	0.0008	0.0015	0.0017	0.0015	0.0013	0.0007	0.0053	0.0049
AY497292	0.2660	0.2669	0.2646	0.2676	0.2676	0.2773	0.2785	0.2799	0.1872	0.1868	0.1872	0.1864	0.0105		0.0013	0.0011	0.0008	0.0008	0.0008	0.0007	0.0007	0.0007	0.0013	0.0015	0.0014	0.0010	0.0008	0.0051	0.0049
azo20	0.2655	0.2678	0.2648	0.2676	0.2678	0.2810	0.2826	0.2838	0.1869	0.1878	0.1871	0.1871	0.0282	0.0283		0.0014	0.0014	0.0013	0.0013	0.0014	0.0014	0.0014	0.0011	0.0011	0.0013	0.0011	0.0015	0.0057	0.0053
DQ399833	0.2660	0.2680	0.2644	0.2690	0.2682	0.2793	0.2807	0.2821	0.1868	0.1861	0.1869	0.1859	0.0225	0.0237	0.0304		0.0011	0.0011	0.0012	0.0013	0.0012	0.0012	0.0015	0.0018	0.0016	0.0008	0.0011	0.0052	0.0048
39mc10	0.2665	0.2677	0.2656	0.2681	0.2688	0.2779	0.2791	0.2795	0.1851	0.1846	0.1853	0.1844	0.0058	0.0082	0.0264	0.0217		0.0006	0.0006	0.0005	0.0005	0.0005	0.0014	0.0015	0.0014	0.0009	0.0005	0.0054	0.0050
178mc10	0.2659	0.2671	0.2637	0.2678	0.2684	0.2775	0.2790	0.2797	0.1852	0.1848	0.1851	0.1846	0.0062	0.0085	0.0259	0.0218	0.0039		0.0005	0.0005	0.0006	0.0006	0.0015	0.0015	0.0015	0.0009	0.0005	0.0053	0.0050
25mc10	0.2665	0.2674	0.2657	0.2679	0.2683	0.2776	0.2791	0.2792	0.1861	0.1857	0.1860	0.1855	0.0060	0.0086	0.0262	0.0215	0.0035	0.0039		0.0005	0.0006	0.0006	0.0013	0.0015	0.0014	0.0011	0.0005	0.0052	0.0048
107mc10	0.2671	0.2686	0.2656	0.2694	0.2700	0.2782	0.2796	0.2800	0.1855	0.1853	0.1855	0.1851	0.0065	0.0090	0.0266	0.0225	0.0044	0.0043	0.0044		0.0006	0.0006	0.0014	0.0016	0.0015	0.0012	0.0006	0.0053	0.0049
136mc10	0.2662	0.2675	0.2642	0.2680	0.2689	0.2772	0.2791	0.2793	0.1858	0.1853	0.1857	0.1851	0.0060	0.0084	0.0268	0.0221	0.0038	0.0038	0.0039	0.0043		0.0005	0.0014	0.0015	0.0014	0.0010	0.0005	0.0054	0.0050
152mc10	0.2645	0.2660	0.2641	0.2671	0.2677	0.2769	0.2781	0.2785	0.1859	0.1854	0.1856	0.1852	0.0061	0.0086	0.0268	0.0222	0.0038	0.0043	0.0040	0.0047	0.0041		0.0014	0.0016	0.0015	0.0011	0.0006	0.0053	0.0050
20mc10	0.2669	0.2690	0.2676	0.2683	0.2682	0.2790	0.2803	0.2812	0.1868	0.1877	0.1867	0.1873	0.0288	0.0287	0.0162	0.0313	0.0268	0.0267	0.0269	0.0273	0.0274	0.0268		0.0008	0.0006	0.0012	0.0015	0.0056	0.0053
115mc10	0.2648	0.2673	0.2674	0.2665	0.2663	0.2790	0.2796	0.2815	0.1865	0.1876	0.1867	0.1869	0.0293	0.0296	0.0162	0.0314	0.0277	0.0278	0.0279	0.0284	0.0283	0.0279	0.0083		0.0008	0.0014	0.0016	0.0056	0.0052
42ori	0.2663	0.2680	0.2664	0.2676	0.2675	0.2798	0.2803	0.2815	0.1867	0.1876	0.1868	0.1869	0.0285	0.0293	0.0159	0.0307	0.0270	0.0268	0.0268	0.0274	0.0272	0.0271	0.0075	0.0055		0.0012	0.0015	0.0057	0.0053
45mc10	0.2668	0.2682	0.2639	0.2692	0.2680	0.2790	0.2804	0.2817	0.1864	0.1857	0.1863	0.1855	0.0223	0.0230	0.0299	0.0081	0.0211	0.0214	0.0209	0.0219	0.0217	0.0215	0.0308	0.0308	0.0299		0.0010	0.0050	0.0047
46mc10	0.2656	0.2668	0.2639	0.2674	0.2680	0.2778	0.2793	0.2794	0.1857	0.1853	0.1854	0.1851	0.0056	0.0083	0.0262	0.0216	0.0035	0.0036	0.0037	0.0042	0.0036	0.0037	0.0266	0.0277	0.0267	0.0211		0.0052	0.0049
NC015993	0.3097	0.3113	0.3089	0.3197	0.3197	0.3148	0.3151	0.3164	0.2664	0.2664	0.2654	0.2668	0.2612	0.2611	0.2611	0.2628	0.2605	0.2610	0.2612	0.2609	0.2614	0.2602	0.2591	0.2581	0.2586	0.2625	0.2601		0.0010
JX486124	0.3111	0.3126	0.3105	0.3199	0.3199	0.3174	0.3176	0.3190	0.2666	0.2667	0.2657	0.2670	0.2636	0.2641	0.2630	0.2658	0.2629	0.2634	0.2637	0.2636	0.2638	0.2626	0.2613	0.2603	0.2608	0.2650	0.2626	0.0091	
