# Supplementary material for: Mitogenomics of recombinant mitochondrial genomes of Baltic Sea Mytilus mussels
Source: Mol Genet Genomics. 2014 Jul 31;289(6):1275–87. doi: 10.1007/s00438-014-0888-3 (PMC4236608; doi:10.1007/s00438-014-0888-3)
Supplement: Supplementary file 3 — Supplementary material 3 (PDF 102 kb) [file 438_2014_888_MOESM3_ESM.pdf]

**Supplementary phylogenetic analyses of the unresolved part of the tree presented in Figure 5**

The analysis involved 11 nucleotide sequences and was conducted in MEGA6 (Tamura et al. 2013). To increase the phylogenetic resolution, the alignement was created containing the continuous section of the genomes in question, beginning at the start of the first *trn* gene (*trnY*) and ending within the *lrn*, immediately before the identified F-M breakpoint. The alignment was checked for potential missed recombination signatures but none was found, therefore this section of the genomes was considered valid for phylogenetic analysis using classic, substitution based models of evolution. The alignment was also straightforward as there were only minor (5 bp in total) length differences, all constituting single nucleotide deletions within the reference NC_006161 genome (most likely sequencing errors, but these columns were excluded from the analyses anyway since all positions containing gaps and missing data were eliminated). There were a total of 15160 positions in the final dataset, 3202 bp more than in the alignment used to obtain the tree presented in Figure 5. In addion to all protein coding genes, this alignment contained also all *trn* genes, *srn* gene, the portion of *lrn* not involved in recombination and all intergenic sequences. The only excluded part was the control region and the part of *lrn* involved in recombination.

**Conclusion:** No increase in the resolution was observed in any analysis (Supplementary Figures S1-S3), as compared to the tree presented in Figure 5.

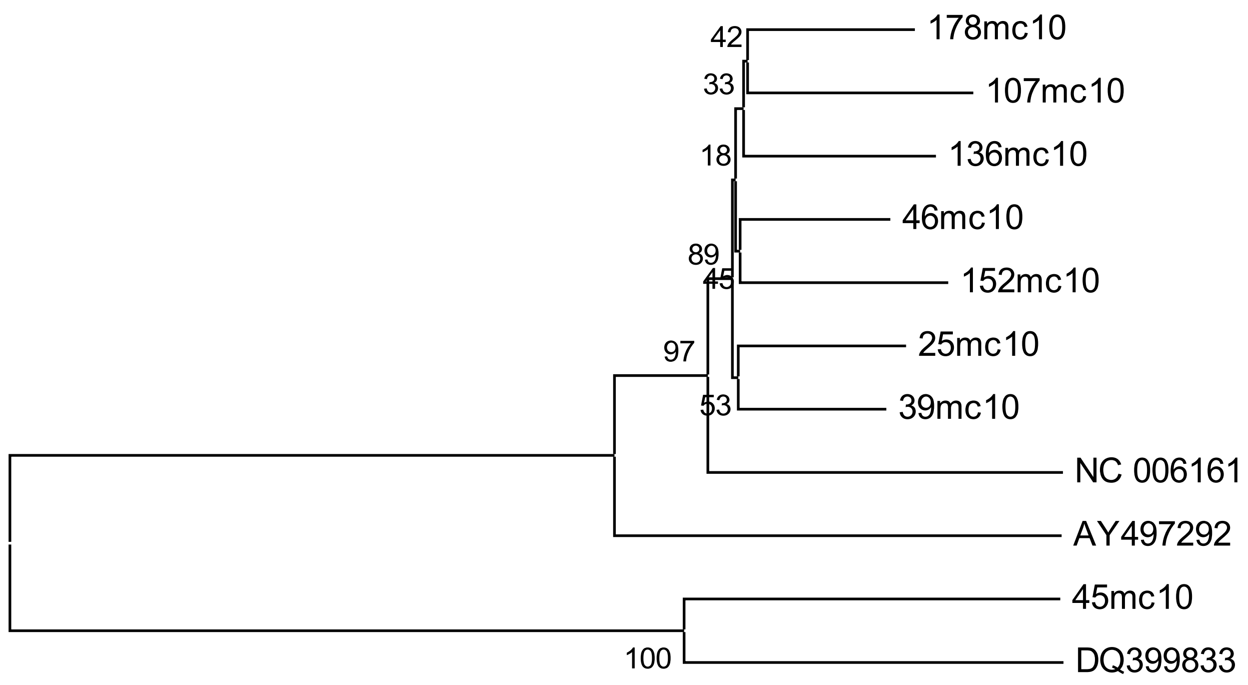


Supplementary Figure S1. Evolutionary relationships of taxa inferred using the Neighbor-Joining method (Saitou and Nei 1987).
The optimal tree with the sum of branch length = 0.03998550 is shown. The percentage of replicate trees in which the associated taxa clustered together in the bootstrap test (100 replicates) are shown next to the branches (Felsenstein 1985). The tree is drawn to scale, with branch lengths in the same units as those of the evolutionary distances used to infer the phylogenetic tree. The evolutionary distances were computed using the Maximum Composite Likelihood method (Tamura et al. 2004) and are in the units of the number of base substitutions per site.

Supplementary Table S3. Model selection for ML (Maximum Likelihood) analysis: fits of 24 different nucleotide substitution models.

| **Model** | **BIC** | **AICc** | ***lnL*** | ***R*** | ***f(A)*** | ***f(T)*** | ***f(C)*** | ***f(G)*** | ***r(AT)*** | ***r(AC)*** | ***r(AG)*** | ***r(TA)*** | ***r(TC)*** | ***r(TG)*** | ***r(CA)*** | ***r(CT)*** | ***r(CG)*** | ***r(GA)*** | ***r(GT)*** | ***r(GC)*** |
|---|---|---|---|---|---|---|---|---|---|---|---|---|---|---|---|---|---|---|---|---|
| TN93+G | 49268.052 | 49017.452 | -24483.722 | 5.65 | 0.272 | 0.348 | 0.143 | 0.236 | 0.023 | 0.009 | 0.082 | 0.018 | 0.202 | 0.015 | 0.018 | 0.491 | 0.015 | 0.094 | 0.023 | 0.009 |
| GTR+G | 49277.348 | 48996.677 | -24470.334 | 4.58 | 0.272 | 0.348 | 0.143 | 0.236 | 0.019 | 0.010 | 0.082 | 0.015 | 0.191 | 0.017 | 0.019 | 0.465 | 0.039 | 0.094 | 0.025 | 0.024 |
| TN93+G+I | 49280.077 | 49019.453 | -24483.722 | 5.65 | 0.272 | 0.348 | 0.143 | 0.236 | 0.023 | 0.009 | 0.082 | 0.018 | 0.202 | 0.016 | 0.018 | 0.491 | 0.016 | 0.094 | 0.023 | 0.009 |
| GTR+G+I | 49289.373 | 48998.678 | -24470.334 | 4.58 | 0.272 | 0.348 | 0.143 | 0.236 | 0.019 | 0.010 | 0.082 | 0.015 | 0.191 | 0.017 | 0.019 | 0.465 | 0.039 | 0.094 | 0.025 | 0.024 |
| TN93 | 49315.694 | 49075.117 | -24513.555 | 5.45 | 0.272 | 0.348 | 0.143 | 0.236 | 0.024 | 0.010 | 0.084 | 0.018 | 0.200 | 0.016 | 0.018 | 0.485 | 0.016 | 0.096 | 0.024 | 0.010 |
| GTR | 49316.985 | 49046.338 | -24496.164 | 4.73 | 0.272 | 0.348 | 0.143 | 0.236 | 0.015 | 0.010 | 0.083 | 0.012 | 0.192 | 0.018 | 0.020 | 0.466 | 0.040 | 0.095 | 0.026 | 0.024 |
| TN93+I | 49327.680 | 49077.080 | -24513.536 | 5.45 | 0.272 | 0.348 | 0.143 | 0.236 | 0.024 | 0.010 | 0.084 | 0.018 | 0.200 | 0.016 | 0.018 | 0.485 | 0.016 | 0.096 | 0.024 | 0.010 |
| GTR+I | 49328.564 | 49047.893 | -24495.942 | 4.73 | 0.272 | 0.348 | 0.143 | 0.236 | 0.015 | 0.010 | 0.083 | 0.012 | 0.192 | 0.018 | 0.020 | 0.466 | 0.040 | 0.095 | 0.026 | 0.024 |
| HKY+G | 49449.617 | 49209.041 | -24580.517 | 5.64 | 0.272 | 0.348 | 0.143 | 0.236 | 0.024 | 0.010 | 0.203 | 0.019 | 0.123 | 0.016 | 0.019 | 0.300 | 0.016 | 0.234 | 0.024 | 0.010 |
| HKY+G+I | 49461.621 | 49211.021 | -24580.507 | 5.65 | 0.272 | 0.348 | 0.143 | 0.236 | 0.024 | 0.010 | 0.203 | 0.019 | 0.123 | 0.016 | 0.019 | 0.300 | 0.016 | 0.234 | 0.024 | 0.010 |
| HKY | 49515.039 | 49284.487 | -24619.240 | 5.43 | 0.272 | 0.348 | 0.143 | 0.236 | 0.025 | 0.010 | 0.202 | 0.020 | 0.123 | 0.017 | 0.020 | 0.298 | 0.017 | 0.233 | 0.025 | 0.010 |
| HKY+I | 49526.897 | 49286.321 | -24619.157 | 5.43 | 0.272 | 0.348 | 0.143 | 0.236 | 0.025 | 0.010 | 0.202 | 0.020 | 0.123 | 0.017 | 0.020 | 0.298 | 0.017 | 0.233 | 0.025 | 0.010 |
| T92+G | 49901.495 | 49680.966 | -24818.480 | 5.62 | 0.310 | 0.310 | 0.190 | 0.190 | 0.022 | 0.014 | 0.163 | 0.022 | 0.163 | 0.014 | 0.022 | 0.266 | 0.014 | 0.266 | 0.022 | 0.014 |
| T92+G+I | 49913.512 | 49682.959 | -24818.476 | 5.62 | 0.310 | 0.310 | 0.190 | 0.190 | 0.022 | 0.014 | 0.163 | 0.022 | 0.163 | 0.014 | 0.022 | 0.266 | 0.014 | 0.266 | 0.022 | 0.014 |
| T92 | 49966.324 | 49755.819 | -24856.907 | 5.43 | 0.310 | 0.310 | 0.190 | 0.190 | 0.023 | 0.014 | 0.162 | 0.023 | 0.162 | 0.014 | 0.023 | 0.264 | 0.014 | 0.264 | 0.023 | 0.014 |
| T92+I | 49978.043 | 49757.514 | -24856.754 | 5.43 | 0.310 | 0.310 | 0.190 | 0.190 | 0.023 | 0.014 | 0.162 | 0.023 | 0.162 | 0.014 | 0.023 | 0.264 | 0.014 | 0.264 | 0.023 | 0.014 |
| K2+G | 50791.026 | 50580.521 | -25269.258 | 5.61 | 0.250 | 0.250 | 0.250 | 0.250 | 0.019 | 0.019 | 0.212 | 0.019 | 0.212 | 0.019 | 0.019 | 0.212 | 0.019 | 0.212 | 0.019 | 0.019 |
| K2+G+I | 50802.746 | 50582.217 | -25269.106 | 5.63 | 0.250 | 0.250 | 0.250 | 0.250 | 0.019 | 0.019 | 0.212 | 0.019 | 0.212 | 0.019 | 0.019 | 0.212 | 0.019 | 0.212 | 0.019 | 0.019 |
| K2 | 50859.838 | 50659.357 | -25309.676 | 5.42 | 0.250 | 0.250 | 0.250 | 0.250 | 0.019 | 0.019 | 0.211 | 0.019 | 0.211 | 0.019 | 0.019 | 0.211 | 0.019 | 0.211 | 0.019 | 0.019 |
| K2+I | 50871.811 | 50661.306 | -25309.650 | 5.42 | 0.250 | 0.250 | 0.250 | 0.250 | 0.019 | 0.019 | 0.211 | 0.019 | 0.211 | 0.019 | 0.019 | 0.211 | 0.019 | 0.211 | 0.019 | 0.019 |
| JC+G | 51462.485 | 51262.003 | -25610.999 | 0.50 | 0.250 | 0.250 | 0.250 | 0.250 | 0.083 | 0.083 | 0.083 | 0.083 | 0.083 | 0.083 | 0.083 | 0.083 | 0.083 | 0.083 | 0.083 | 0.083 |
| JC+G+I | 51474.509 | 51264.004 | -25610.999 | 0.50 | 0.250 | 0.250 | 0.250 | 0.250 | 0.083 | 0.083 | 0.083 | 0.083 | 0.083 | 0.083 | 0.083 | 0.083 | 0.083 | 0.083 | 0.083 | 0.083 |
| JC | 51522.906 | 51332.449 | -25647.222 | 0.50 | 0.250 | 0.250 | 0.250 | 0.250 | 0.083 | 0.083 | 0.083 | 0.083 | 0.083 | 0.083 | 0.083 | 0.083 | 0.083 | 0.083 | 0.083 | 0.083 |
| JC+I | 51534.889 | 51334.408 | -25647.201 | 0.50 | 0.250 | 0.250 | 0.250 | 0.250 | 0.083 | 0.083 | 0.083 | 0.083 | 0.083 | 0.083 | 0.083 | 0.083 | 0.083 | 0.083 | 0.083 | 0.083 |

NOTE.-- Models with the lowest BIC scores (Bayesian Information Criterion) are considered to describe the substitution pattern the best. For each model, AICc value (Akaike Information Criterion, corrected), Maximum Likelihood value (lnL), and the number of parameters (including branch lengths) are also presented (Nei and Kumar 2000). Non-uniformity of evolutionary rates among sites may be modeled by using a discrete Gamma distribution (+G) with 4 rate categories and by assuming that a certain fraction of sites are evolutionarily invariable (+I). Assumed or estimated values of transition/transversion bias (R) are shown for each model. They are followed by nucleotide frequencies (f) and rates of base substitutions (r) for each nucleotide pair. Relative values of instantaneous r should be considered when evaluating them. For simplicity, sum of r values is made equal to 1 for each model. For estimating ML values, a tree topology was automatically computed.

Abbreviations: GTR: General Time Reversible; HKY: Hasegawa-Kishino-Yano; TN93: Tamura-Nei; T92: Tamura 3-parameter; K2: Kimura 2-parameter; JC: Jukes-Cantor.

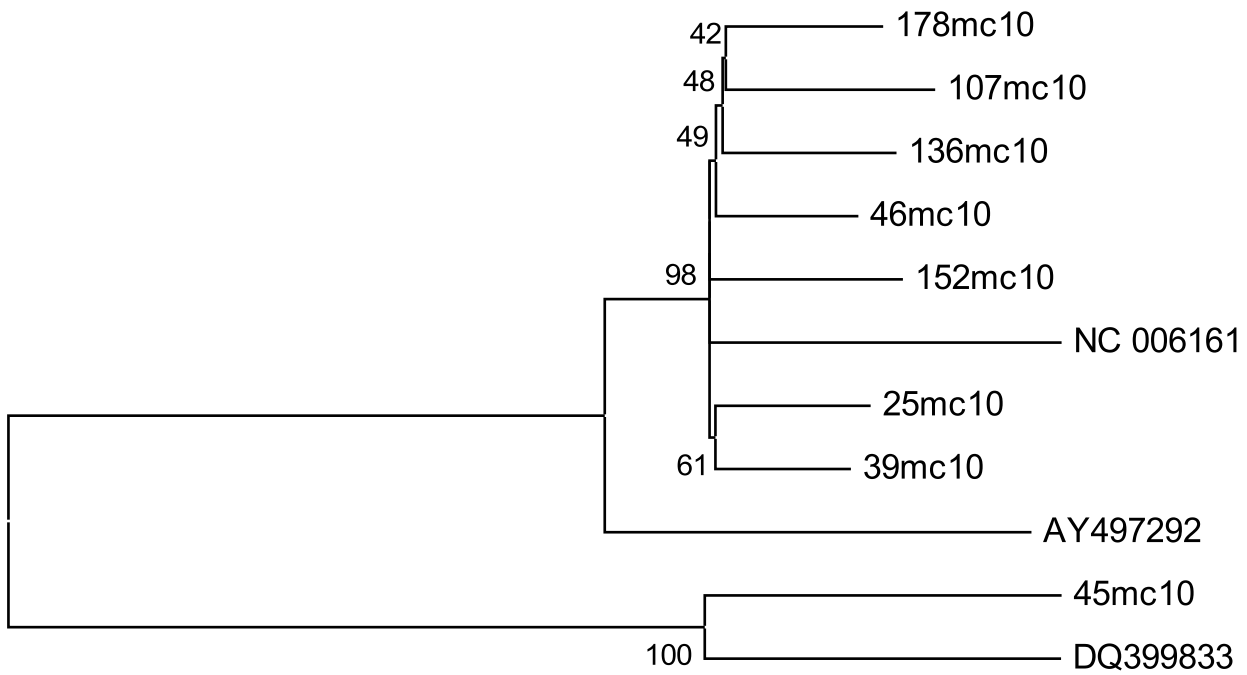


Supplementary Figure S2. Molecular Phylogenetic analysis by Maximum Likelihood (ML) method.
The evolutionary history was inferred by using the Maximum Likelihood method based on the Tamura-Nei model (Tamura and Nei 1993), selected based on Bayesian Information Criterion (Supplementary Table S3). The tree with the highest log likelihood (-24480.3394) is shown. The percentage of trees in which the associated taxa clustered together is shown next to the branches. Initial trees for the heuristic search were obtained automatically by applying Neighbor-Join and BioNJ algorithms to a matrix of pairwise distances estimated using the Maximum Composite Likelihood (MCL) approach, and then selecting the topology with superior log likelihood value. A discrete Gamma distribution was used to model evolutionary rate differences among sites (4 categories (+G, alpha parameter = 0.0905)). The tree is drawn to scale, with branch lengths measured in the number of substitutions per site.

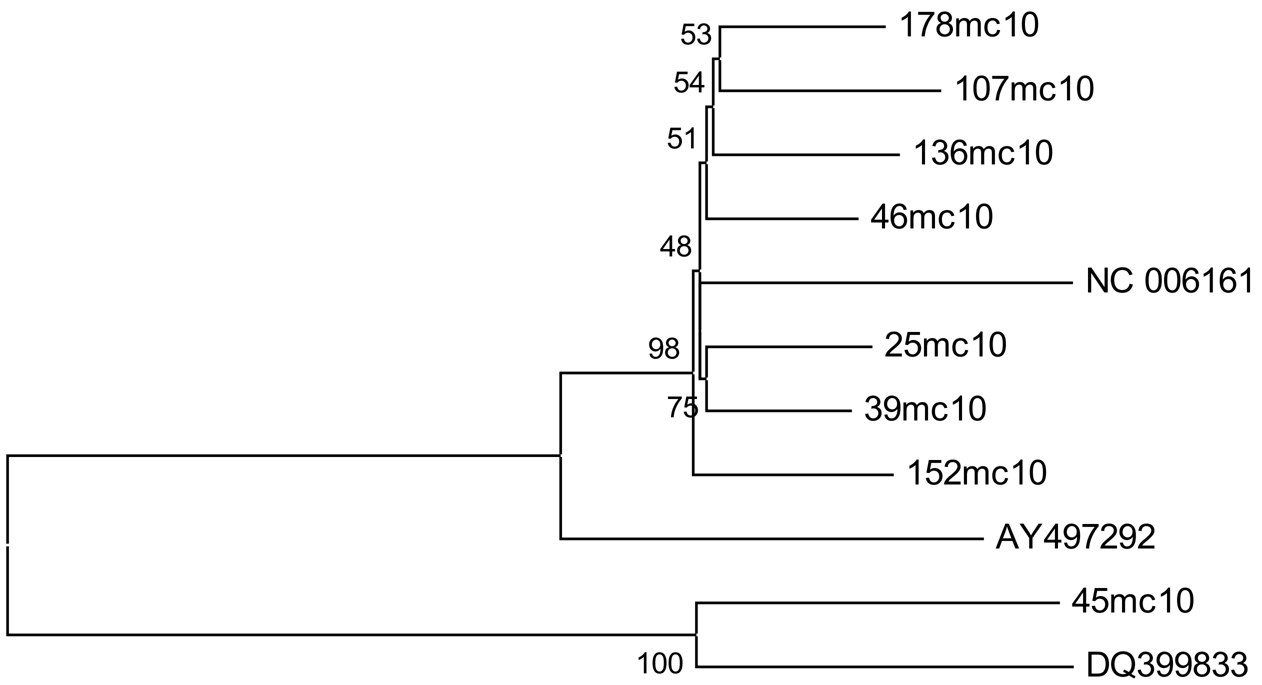


Supplementary Figure S3. Maximum Parsimony (MP) analysis of taxa
The evolutionary history was inferred using the Maximum Parsimony method. Tree #1 out of 3 most parsimonious trees (length = 605) is shown. The consistency index is ( 0.865613), the retention index is ( 0.869231), and the composite index is 0.820381 ( 0.752417) for all sites and parsimony-informative sites (in parentheses). The percentage of replicate trees in which the associated taxa clustered together in the bootstrap test (1000 replicates) are shown next to the branches (Felsenstein 1985). The MP tree was obtained using the Tree-Bisection-Regrafting (TBR) algorithm (Nei and Kumar 2000, pg. 126) with search level 5 in which the initial trees were obtained by the random addition of sequences (30 replicates). The tree is drawn to scale , with branch lengths calculated using the average pathway method (Nei and Kumar 2000, pg. 132) and are in the units of the number of changes over the whole sequence.

**Supplementary references for phylogenetic analysis.**


Felsenstein J. (1985) Confidence limits on phylogenies: An approach using the bootstrap. Evolution 39:783-791.

Nei M. and Kumar S. (2000) Molecular Evolution and Phylogenetics. Oxford University Press, New York.

Saitou N. and Nei M. (1987) The neighbor-joining method: A new method for reconstructing phylogenetic trees. Molecular Biology and Evolution 4:406-425.

Tamura K. and Nei M. (1993) Estimation of the number of nucleotide substitutions in the control region of mitochondrial DNA in humans and chimpanzees. Molecular Biology and Evolution 10:512-526.

Tamura K., Nei M., and Kumar S. (2004) Prospects for inferring very large phylogenies by using the neighbor-joining method. Proceedings of the National Academy of Sciences (USA) 101:11030-11035.

Tamura K., Stecher G., Peterson D., Filipski A., and Kumar S. (2013) MEGA6: Molecular Evolutionary Genetics Analysis version 6.0. Molecular Biology and Evolution30: 2725-2729.
